# The impact of interprofessional task-based training on the prevention of surgical site infection in a low-income country

**DOI:** 10.1186/s12909-021-03046-3

**Published:** 2021-12-09

**Authors:** Muhammad Nasir Ayub Khan, Daniëlle M. L. Verstegen, Asma Shahid, Diana H. J. M. Dolmans, Walther Nicolaas Anton van Mook

**Affiliations:** 1 Department of Health Professions Education, Shifa International Hospital and Shifa Tameer-e-Millat University, Islamabad, Pakistan; 2 School of Health Professions Education, Maastricht University, Maastricht, The Netherlands; 3 Department of Anesthesia, Shifa International Hospital, Islamabad, Pakistan; 4 Department of Intensive Care Medicine, Maastricht University Medical Center, Maastricht, The Netherlands

**Keywords:** Surgical site infection, Self-perceived behavior, Operating rooms, Task-based interprofessional training

## Abstract

**Background:**

Training is considered instrumental in reducing surgical site infection. We developed training based on authentic tasks, interprofessional learning, and reflective learning for implementation in a low-income country where such training opportunities are rare. This study evaluated the results of training in terms of participants’ acceptance, participants’ knowledge acquisition, and their self-perceived behavior change.

**Methods:**

We included 145 participants in the voluntary training program, comprising 66 technologists (45.5%), 43 nurses (29.7%), and 36 doctors (24.8%) from Shifa International Hospital, Islamabad, Pakistan. We measured “satisfaction” using a questionnaire at the end of the training, “knowledge” through pre-and post-intervention assessments, and “self-perceived behavior change” using a questionnaire and interviews 8 weeks post-training.

**Results:**

Pre- and post-test scores showed a significant increase in knowledge. Participants were favorable to the training and eager to participate. They positively applied in practice what they had learned about preventing surgical site infection. Our qualitative data analysis revealed two categories of themes, representing the upsides of the training as it stood, and existing factors or downsides that hindered the effective transfer of learning to practice.

**Conclusion:**

Participants were very enthusiastic about the training format. The knowledge test showed a gain in knowledge. Moreover, participants acknowledged that their behavior toward the prevention of surgical site infection in the operating rooms had changed. The use of authentic tasks from daily clinical practice, as well as the interprofessional approach and reflection, were considered to promote the transfer of learning. Although promising, our findings also pointed to obstacles limiting the application of evidence-based knowledge, such as a shortage of supplies and conventional practices.

**Supplementary Information:**

The online version contains supplementary material available at 10.1186/s12909-021-03046-3.

## Background

Surgical site infection is one of the most common hospital-associated infections. In an unfortunate turn of events, it poses a threat to patient safety because of the high incidence of morbidity and mortality associated with it [1]. It accounts for 20% of all health-associated infections among hospitalized patients in the developed world. In low-income countries, moreover, its incidence is even 20 times higher compared to higher-income countries [1]. The burden of surgical site infection in these countries can be attributed, at least in part, to a lack of knowledge and training to prevent it [1, 2]. The World Health Organization (WHO), the Association for Professionals in Infection Control, the Center for Disease Control and Prevention, the Society for Healthcare Epidemiology of America, and the Institute of Health Improvement have emphasized the importance of training healthcare professionals working in the operating rooms to prevent surgical site infections [3].

Similarly, previous research has revealed that the training of healthcare professionals is instrumental in reducing this problem [4, 5]. We therefore designed training based on the principles of authentic tasks [6, 7], interprofessional training [8, 9], and reflective learning [10]. To enhance the application and transfer of knowledge to healthcare workers’ practice in the operating room, we ensured that the training context was similar to the real hospital setting. Participants were presented with authentic learning tasks in an interprofessional group of trainees, such that they also worked within the practice. Reflective learning was stimulated because it helped participants evaluate and compare their actual learning with good practice to prevent surgical site infections. Task-based training has previously been successfully applied in different health professional settings, such as teaching immunohistochemistry to postgraduates in China [11], continuing medical education in neurology in Pakistan [6], and in the undergraduate medical clinical years in Turkey [12]. As most institutions in a low-income country rely on traditional teaching methods that emphasize passive knowledge acquisition and rote memorization with limited relevance to clinical tasks and healthcare professionals [13], however, implementing the said training could be challenging.

We evaluated the training on a small scale by collecting the experiences of both participants and trainers. However, we did not evaluate the impact of the training with larger groups of participants. Based on our preliminary evaluation results, we improved the training design, for instance by adding an extra session at the end of the training, as suggested by participants. The purpose of this additional session was to discuss participants’ experiences of using what they had learned in practice. The key focus was on discussing what went well and what did not about preventing surgical site infection in the operating rooms. Intended to strengthen the training’s design principles, the session enhanced the transfer of learning to practice: Participants discussed their experience (authentic learning) with their colleagues (interprofessional learning) and reflected on barriers and ways to overcome these (reflective learning).

The present study aimed to evaluate this revised, task-based, interprofessional training program on the prevention of surgical site infection in a low-income country. More specifically, we used a mixed-methods concurrent approach to explore participants’ satisfaction with the training, measure its effects on the acquisition of knowledge, and investigate participants’ self-perceived behavior change.

### Research questions

1 What are participants’ perceptions of and satisfaction with the training? How well was the intervention implemented? (Questionnaire 1, completed at the end of the last training session).

2 Does participants’ knowledge about the prevention of surgical site infection increase after the training? (pre-and post-training assessments).

3 How do participants perceive potential behavioral changes following the infection prevention training program? (Questionnaire 2 and interview with participants: completed for evaluation of self-perceived behavior 8 weeks after training).

## Methods

### Study design

We conducted a mixed-methods study using a concurrent design to evaluate the perceived and measured effectiveness of a design-based, interprofessional, task-based training that had been previously described, piloted, revised, and subsequently implemented. After redesigning the original training based on participants’ feedback and suggestions [14], we implemented the new training on a larger scale but in the same context.

The study was set at Shifa International Hospital, a jointly commissioned, internationally accredited, private tertiary care hospital of Shifa Tameer-e-Millat University in Islamabad, Pakistan, that is home to undergraduate and postgraduate medical and nursing schools. It is a postgraduate training center for many specialties, recognized by the college of physicians and surgeons of Pakistan, with a 650-bed hospital and 24 operating rooms performing more than 1500 surgeries per month.

### Intervention

An educational intervention based on the principles of whole task-based, interprofessional, reflective learning was designed and implemented in the operating rooms to acquired knowledge and induce change in behavior regarding the prevention of surgical site infection. The training content addressed the 2016 global guidelines for the prevention of surgical site infections by the WHO. Spanning five workshops of 1 h and 30 min each held consecutively on Wednesdays or Thursdays, the training lasted 7.5 h in total. The training was conducted by three experienced and trained facilitators from the nursing education department and the anesthesia department, respectively, each with extensive experience of conducting problem-based learning at the undergraduate level at Shifa Tameer-e-Millat University.

### Participants

Participants were 145 healthcare professionals from the operating rooms, including 66 technologists (45.5%), 43 nurses (29.7%), and 36 doctors (24.8%) (Table 1). They enrolled voluntarily after an invitation to take part in the training sent via email and WhatsApp groups by the operating room nurse educator. Interested respondents enrolled after providing informed, written consent. Approximately 8 weeks after the training, we approached all training participants for an interview by email. The first author of this study interviewed the first 10 who accepted the invitation.

**Table 1:** Background of the participants

*Technologists 66 (45.5%):* The technologists were diploma holders from the Faculty of Health Sciences who had been certified after completion of an approved two-year training course. The vital members of the team were surgical technologists who ensured safe surgical treatment and avoided contamination of the surgical site. They prepared patients for surgery before the operative procedure by washing, shaving, and disinfecting the site of the surgical incision. *Nurses 43 (29.7%):* The nurses had completed a four-year bachelor’s degree in nursing and were registered with the Pakistan Nursing Council. They were responsible for following and enforcing strict standards of aseptic techniques and infection prevention procedures in operating rooms, such as ensuring compliance with hand hygiene. *Doctors 36 (24.8%):* The doctors were anesthesia and surgical residents who had passed their primary fellowship exam of the College of Physicians and Surgeons in Anesthesia and Surgery. At the time of training, they were practicing their postgraduate training in their respective fields.	

### Instruments for data collection

**Fig. 1:**
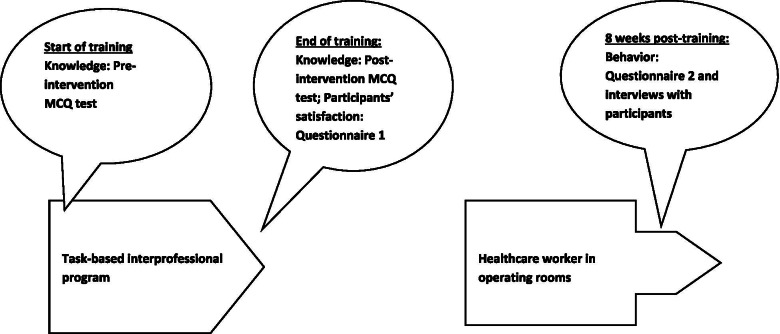
The process of evaluating the implementation of task-based, interprofessional training in a low-income country: Pakistan. Knowledge was evaluated through pre-and post-intervention MCQ tests, participants’ satisfaction through questionnaire 1 at the end of the training, and self-perceived behavior change through questionnaire 2 and interviews eight weeks post-training

We wielded the following instruments to evaluate the three levels of Kirkpatrick [15]; level 1 satisfaction with the training, level 2 knowledge acquired, and level 3 self-perceived behavioral changes, as shown in Fig. 1.

#### Participants’ satisfaction with the training

At the end of the last training session, we measured participants’ satisfaction with the training using *questionnaire 1* which addressed the first research question shown in Fig. 1. We operationalized “satisfaction” as the extent to which participants were satisfied with the training content and learning environment [15]. The questionnaire consisted of 16 items on a 5-point Likert scale ranging from strongly disagree (1) to strongly agree (5) that probed into how participants felt, what they thought, and how they experienced the training.

#### Knowledge

We used *pre-and post-intervention knowledge assessments* to measure participants’ knowledge acquisition, addressing research question 2 shown in Fig. 1. Consisting of 14 multiple-choice questions (MCQs) about the training content, this test measured basic knowledge, the application of knowledge, as well as analytical and synthesizing skills. The MCQs were prepared by two educationalists, pilot-tested on non-participants, and reviewed by an expert panel before use in this study.

#### Self-perceived behavioral change

We operationalized self-perceived behavioral change as the transfer of what was learned during the training to job performance. First, we administered a questionnaire (*questionnaire 2*) to collect information on self-perceived behavioral change 8 weeks post-training shown in Fig. 1 [10]. This questionnaire consisted of 10 items on a 5-point Likert scale ranging from strongly disagree (1) to strongly agree (5) that probed into the extent to which participants applied in the operating rooms what they had learned during the training. Next, we held 10 *semi-structured individual interviews with participants* 8 weeks following the training. Interviews lasted 15 min and were conducted by the primary researcher using a guide to gain insight into the changes in participants’ behavior to prevent surgical site infection. The ultimate aim was to gain insight into how the training had made them change their practice or how it had not (and why). The interview guide is included in Additional file 1: Appendix 1.

### Data analysis

We used Statistical Package for the Social Sciences (SPSS) software (IBM Corp, Released 2013, IBM SPSS Statistics for Windows, Version 22.0 Armonk, NY: IBM CORP) to obtain descriptive statistics for the analysis of our quantitative data, including *means (M)* and *standard deviations (SD)* for each item. We also ran t-tests to compare the pre-and post-test results.

Two authors (MNA and AS) independently read the qualitative data, defining common patterns using a continuous comparative approach based on the commonly accepted concepts of primary, secondary, and tertiary coding, the identification of trends, and the use of participants’ shared opinions [16]. The topics identified were coded independently to allow for a comparison of participants’ responses. Before consensus was reached, all differences were addressed. These themes were presented to the second, fourth, and fifth authors during face-to-face online meetings via Skype. To test assumptions, the authors challenged the themes and illustrative codes. The Results section will feature illustrative quotes.

### Ethical approval

We obtained approval from the ethical committee of Shifa Tameer-e-Millat University (reference number IRB#329–819 = 2019). Participants could withdraw at any time for any reason. The data handling and storage were compatible with the law.

## Results

In the next paragraphs, we will discuss the quantitative and qualitative results of this study consecutively**.**

### Quantitative results

#### Questionnaire 1: participants’ satisfaction with the training program

Table 2 shows the mean scores (and standard deviations) for the factors relating to participants’ satisfaction with the training. All items had high mean scores (i.e., > 4) on a 1–5 scale. This holds for all the five scales, spanning goal achievement, the use of authentic tasks, how the facilitators supported the participants, the interprofessional characteristics of the training, and the organization. Hence, participants were generally very positive about all aspects of the training.

**Table 2:** Evaluation of participants’ satisfaction with the training program (*N* = 145)

Serial No.	Item	Mean (1 to 5)	SD ±
**Theme 1: Goal achievement**			
1	The training helped me understand why it is essential to stick to surgical site infection guidelines related to operating rooms	4.62	0.50
2	The training helped to gain knowledge of surgical site infection prevention	4.40	0.49
3	This training helped to reflect on behavior regarding infection prevention	4.58	0.52
4	During this training, I learned to speak up to colleagues to prevent infections	4.60	0.49
**Theme 2: Use of authentic tasks**			
5	The task used was relevant to day-to-day clinical practice to prevent infections in the operating rooms	4.69	0.54
6	The video presentation was helpful to understand good practice to prevent surgical site infections	4.63	0.49
7	The video stimulated interest in infection prevention	4.61	0.51
8	The reading materials were helpful to gain a better understanding of infection prevention	4.57	0.59
9	The variety of tasks used in this training was helpful to gain a better understanding	4.69	0.46
**Theme 3: The facilitators**			
10	Facilitators encouraged learners to participate actively in training	4.62	0.53
11	The facilitators stimulated discussion among participants	4.63	0.49
12	The facilitators gave us full feedback	4.56	0.55
13	The facilitators stimulated participants to reflect on their practice	4.55	0.52
**Theme 4: Interprofessional learning**			
14	Discussion with professionals from different backgrounds helped to gain a better understanding of each other’s perspectives	4.45	0.55
15	Discussions with professionals from different backgrounds were helpful to learn to speak up to other professionals	4.45	0.55
16	I learned to provide constructive feedback to other health professionals	4.51	0.55
17	Learning with other professionals during this training was helpful to understand that intervention requires teamwork	4.57	0.49
**Theme 5: Organization**			
18	The training was well organized	4.70	0.44

##### Pre-test and post-test knowledge scores

Table 3 shows a significant difference between pre-test and post-test scores with *p* < 0.00. The mean pre-test score was 49.10, and the mean post-test score was 86.46 (on a 1–100 scale).

**Table 3:** Pre-test and post-test scores

Test scores	Minimum	Maximum	Range	N	Mean	SD	***P***-value
**Pre-test score**	10	100	90	145	49.10	±8.02	< 0.00
**Post-test score**	40	100	60	145	86.46	±14.06	

### Questionnaire 2: participants’ self-perceived change in behavior after the training

Table 4 shows the mean scores (and standard deviations) for the three factors and their items relating to participants’ self-perceived behavior change after the training. All items’ scores were high (i.e., > 4.1 on a 1–5 scale).

**Table 4:** Self-perceived behavior change (*N* = 145)

Serial No.	Item	Mean (1 to 5)	SD ±
**Theme 1: Application of knowledge**			
1	I applied what I learned about infection prevention during the training in my daily work	4.11	0.66
2	After the training, I became stricter in following infection prevention guidelines	4.31	0.58
3	After the training, I became better able to deal with barriers related to infection prevention	4.24	0.65
4	After the training, I became better able to speak up to others about infection prevention	4.26	0.70
**Theme 2: Teamwork and collaboration**			
5	After the training, I had a better understanding of other team members’ roles in infection prevention	4.26	0.67
6	I have become more effective as a team member in infection prevention	4.33	0.62
7	Working relationships improved about playing a role in infection prevention	4.26	0.65
8	I now communicate in a better way with other healthcare professionals in the operating room	4.22	0.66
**Theme 3: Roles and responsibilities**			
9	I speak up to others about infection prevention when necessary	4.40	0.61
10	After the training, I had a better understanding of the professional role in infection prevention	4.40	0.67

### Qualitative results

Our qualitative analysis of the interviews resulted in the identification of two overarching themes that represented the upsides of the training as it stood and the downsides or factors that hindered the effective transfer of the things learned to practice.

#### Theme 1: the upsides of the current training

The following sub-themes represent the positives of the training in its current format. Illustrative quotes are provided wherever applicable*.*

##### The training produced a positive change

Participants believed that the training increased their understanding and knowledge, which they could subsequently apply in the operating rooms to prevent infection:

*“This is the operating room; People are more considerate now about infection prevention. For instance, a few days ago, the use of razors in the operating room was met with criticism.“.*

##### The training produced the will to overcome barriers

Participants reported that the training had motivated them to follow the guidelines by making them understand the weakness of their healthcare system. Moreover, it had provided them with the will to overcome the barriers that hindered the application of knowledge to prevent surgical site infection:

*“Whenever someone tries to initiate an innovative task, there are hurdles. However, you just need to have the will and take the lead. It is because barriers are just in* [your] *mind. So if one overcomes them, it is a win-win situation.”*

##### The training includes relevant tasks from day-to-day practice

Participants considered the tasks used during the training as relevant to the day-to-day practice of preventing infection. The use of these tasks during the training helped to gain a deeper understanding of how to apply good practice to prevent surgical site infection in the operating rooms:

*“The tasks assigned to us were the best part of the training as they were routine practice for us that we take to prevent surgical site infections. By solving them, we got a deeper insight, but we also learned to communicate better with other professionals about the solution. Along with this, it also gave me practical answers to my doubts and queries.”*

##### The multidisciplinary approach improved understanding of one’s role and of that of other healthcare professionals

Many participants felt that the training had helped them to take a leading role, and to understand their responsibilities in preventing surgical site infection in the operating rooms**:**

*“Prevention of surgical infection is not a one-man show. It requires a multidisciplinary approach by involving the mutual coordination of healthcare professionals. Hence, working as a team helped us improve our communication and enabled us to formulate new ways to overcome the obstacles and ensure safe practice for the patients.”*

#### Theme 2: the downsides of the current training

The following sub-themes represent the downsides of the current training that hindered the effective implementation of the things learned in the operating rooms. Illustrative quotes are provided wherever applicable*.*

##### Turnover of healthcare professionals complicates effective transfer of learning

Some of the participants suggested that the frequent turnover of healthcare professionals complicated the application of the guidelines in everyday practice, often making it more “tricky.” Indeed, health professionals are continually leaving jobs to search for better financial opportunities elsewhere:

*“Health professionals leave jobs regularly because* [of which] *newcomers are not connected to the team; Therefore, this training should be provided more often so that they can help with the team.”*

##### Shortage of supplies hinders the implementation of guidelines

Some participants indicated that, despite the best of training, they could not follow the guidelines because the lack of medical supplies forced them to use alternative means, which, moreover, were not supported by scientific evidence:

*“Everybody says avoid razors, but because we could have clippers for five months and* [then] *have no clippers for a month, ..we are forced to go* [over] to razors.”

##### Conventional practice hinders the implementation of guidelines

Several participants noticed that some senior surgeons were guided by their personal opinion rather than by scientific evidence when engaging in clinical practice. That is, they preferred to stick to their conventional practices:

*“The infection prevention recommendations can also be difficult to use in practice as plastic surgeons also prefer to use razors to provide a hairless skin graft in plastic surgery.”*

## Discussion

This research was designed to evaluate the outcome of a task-based training aimed to prevent surgical site infection in the operating rooms that were implemented in a low-income country. In researching participants’ perceptions and acceptance of this training, we found that participants were pleased with how the training was conducted. They reported that their behavior in the operating rooms had improved at about 8 weeks after the training (self-perceived behavior change). Moreover, participation in the training led to a knowledge gain, as was demonstrated by the improved scores for the post-test compared to pre-test scores. Subsequent interviews with the participants made clear that the use of tasks from daily infection control practices in the operating rooms and discussion with other healthcare professionals facilitated the transfer of learning to practice. Similarly, the use of authentic tasks and reflection on how participants’ current practice compared to recommended practices to prevent surgical site infection was perceived to promote the transfer of learning. Although the quantitative data showed positive results, the interview data pointed to some hurdles, such as the frequent turnover of professionals, a lack of supplies, and difficulties to break with conventional practices.

Participants were also positive about the fact that learning took place in small interprofessional groups. Promoting active learning, this approach stimulated them to reflect on ways to prevent surgical site infections [9, 11]. Participants were placed in complex situations for them to analyze and learn how to solve these together. As the training differed from the conventional teaching methods often used in health institutions in Pakistan, such as lecturing [17], most participants had their first experience of being actively engaged in learning tasks. More specifically, through brainstorming, self-study, and discussions, participants were actively involved in discussing the tasks assigned to them to avoid surgical site infection. These activities, combined with authentic, professionally relevant tasks, are perceived to induce behavioral changes in participants [11–13].

Another reason why this interprofessional learning was perceived as beneficial for the transfer of learning was that the group composition resembled that of the teams in which participants worked in real practice. The small groups enabled participants to gain insight into the strengths and weaknesses of the healthcare system they worked in by reflecting on their current infection control practices. Videos showing good practices to prevent surgical site infection served as input for discussion. Finally, participants discussed strategies to overcome the flaws of the system and reflected on ways to reduce surgical site infection and improve patient safety. This training was perceived to improve the behavior of the participants.

### Strengths and limitations

The strength of this study is that the training was based on current instructional design principles, including the use of authentic, meaningful tasks, interprofessional teams, as well as reflection. The strength of this study is that we used various tools to evaluate the training, both at the end of and 8 weeks after the training.

A few limitations also need addressing. The first limitation is that this is a single-center study. The training was implemented in a private hospital in a resource-poor setting, limiting the transferability of the results to the public sector hospitals where resources may be even scarcer than in the private hospital where this study was performed. A second limitation is that the training was of relatively short duration, which might not have been enough to effect a sustainable change in preventing surgical site infection in the operating rooms. If such change is desired, institutions and countries should develop a master plan to ensure repeated training to prevent surgical site infections in low-income countries. A third limitation is that although changes in pre−/post- knowledge tests results have been found, this provides insufficient evidence for determining that these can primarily be attributed to the training or a re-testing effect We have no further data about the construct validity of the test, but we ensured the content validity given that the test items were measuring four aspects we included in the training basic knowledge about infection, application of knowledge into clinical practice of infection prevention. A fourth limitation of this study is that the positive responses might be partly caused by participants giving socially desirable answers. Participants did, however, also mention negative aspects of the training during interviews and the researchers stimulated them to take a critical. A final limitation is that we only collected self-reported data, without observing how participants behaved in real practice.

### Implications for research

Future research should focus on the effect of the training on the incidence of surgical site infection at the institutional level, coupled with measurements and observations of the mid-and long-term outcome in terms of increased compliance with infection guidelines. We also invite replications of our study in other hospitals with a similar or different context. Further observational studies are needed to investigate the effects of longitudinal training on the behavioral changes in healthcare professionals to prevent surgical site infection.

### Implications for practice

This interprofessional task-based training should be provided to all healthcare professionals at the beginning of their job and periodically, at least every 2 years, to prevent surgical site infection. Periodic evaluation of the effectiveness of the training program and assessment of knowledge of preventing infection in operating rooms should be undertaken on a routine basis. As a minimum requirement for the renewal of their job contracts, healthcare professionals should be requested to participate in training to prevent surgical site infection.

## Conclusion

The training was well-received by participants and led to a significant knowledge gain as the difference between pre-test and post-test scores suggested. The discussion with other healthcare practitioners while solving authentic tasks, coupled with current, good practices to prevent surgical site infection enabled participants to practice what was taught during the training. The use of authentic tasks from daily clinical practice was considered to promote the transfer of knowledge to practice. However, although the findings from our quantitative survey were very promising, the interview results showed that there are some obstacles to the application of evidence-based knowledge to prevent surgical site infections, such as the frequent turnover of healthcare professionals, a lack of supplies, and difficulties to break with conventional practices.

## Supplementary Information

**Additional file 1 Appendix 1**. Semi-structured interview guide.

## Data Availability

The data generated is not publically available due to confidentiality clauses but the authors declare there no competing interest’s anonymized versions available from the corresponding author on reasonable request.

## Abbreviation

WHO: World health organization

## References

[1] Rojas-Gutierrez E, Vilar-Compte D. An overview of surgical site infection in low-and middle-income countries: the role of recent guidelines, limitations, and possible solutions. Curr Treat Options Infect Dis. 2019;11(3):300–16.

[2] Khan MN, Verstegen DM, Bhatti AB, Dolmans DH, van Mook WN (2018). Factors hindering the implementation of surgical site infection control guidelines in the operating rooms of low-income countries: a mixed-method study. Eur J Clin Microbiol Infect Dis.

[3] Aucamp MC. Best practices for teaching healthcare workers about infection prevention and control: a systematic review (Doctoral dissertation, Stellenbosch: Stellenbosch University).

[4] Safdar N, Abad C (2008). Educational interventions for the prevention of healthcare-associated infection: a systematic review. Crit Care Med.

[5] Cherry MG, Brown JM, Neal T, Ben SN (2010). What features of educational interventions lead to competence in aseptic insertion and maintenance of CV catheters in acute care? BEME guide no. 15. Med Teach.

[6] Susilo AP, van Merriënboer J, van Dalen J, Claramita M, Scherpbier A (2013). From lecture to learning tasks: use of the 4C/4D model in a communication skills course in a continuing professional education context. J Contin Ed Nurs.

[7] Dolmans DH, Wolfhagen IH, Van Merriënboer JJ (2013). Twelve tips for implementing whole-task curricula: how to make it work. Med Teach.

[8] Flood B, Smythe L, Hocking C, Jones M (2019). Interprofessional practice: beyond competence. Adv Health Sci Educ.

[9] Vijn TW, Wollersheim H, Faber MJ, Fluit CR, Kremer JA (2018). Building a patient-centered and interprofessional training program with patients, students and care professionals: study protocol of a participatory design and evaluation study. BMC Health Serv Res.

[10] Wang Q, Li H, Pang W, Liang S, Su Y (2016). Developing an integrated framework of problem-based learning and coaching psychology for medical education: participatory research. BMC Med Educ.

[11] Tian Y, Li C, Wang J, Cai Q, Wang H, Chen X, Liu Y, Mei F, Xiao L, Jian R, Li H (2017). Modified task-based learning program promotes problem-solving capacity among Chinese medical postgraduates: a mixed quantitative survey. BMC Med Educ.

[12] Vakani F, Jafri W, Ahmad A, Sonawalla A, Sheerani M (2014). Task-based learning versus problem-oriented lecture in neurology continuing medical education. JCPSP.

[13] Ozkan H, Degirmenci B, Musal B, Itil O, Akalin E, Kilinc O, Ozkan S, Alici E (2006). Task-based learning program for clinical years of medical education. Educ Health.

[14] Dolmans DH, Tigelaar D (2012). Building bridges between theory and practice in medical education using a design-based research approach: AMEE guide no. 60. Med Teach.

[15] Moldovan L (2016). Training outcome evaluation model. Procedia Technol.

[16] Thomas DR. A general inductive approach for qualitative data analysis.

[17] Tracy SJ. Qualitative quality: Eight “big-tent” criteria for excellent qualitative research. Qual Inq. 2010;16(10):837–51.

[18] Latif MZ, Nizami R, Riaz H. Faculty perceptions about continuing medical education activities. Adv Health Prof Educ. 2016;2(1):16–9.

