# Supplementary material for: The impact of interprofessional task-based training on the prevention of surgical site infection in a low-income country
Source: BMC Med Educ. 2021 Dec 9;21:607. doi: 10.1186/s12909-021-03046-3 (PMC8656023; doi:10.1186/s12909-021-03046-3)
Supplement: Supplementary file 1 — Additional file 1 Appendix 1. Semi-structured interview guide. [file 12909_2021_3046_MOESM1_ESM.docx]

**Appendix 1: Semi-structured interview guide**

**Introduction: I would like to ask you some questions regarding the SSI training that you followed a few weeks ago. I will use your feedback to improve this training further, so please feel free to share positive and less positive experiences. What you tell me will only be reported anonymously.**

***Opening question***

1. What do you remember most about the training?

***Main questions***

1. We are especially interested in whether you have been able to apply what you have learned in practice.

2. What are your experiences trying to apply what you learned during the practice course?

3. What did you or your team change? Can you give some examples? Can you explain how you introduced this change?

4. Which characteristics or aspects of the course were helpful to make this change happen? Can you give an example and explain why this was helpful?

5. What could you not do in practice? Why?

6. What are the barriers that you meet when you try to apply what you learned?

7. During the training, you worked in a group of professionals with different training backgrounds (physicians, nurses, technicians). How did this enhance you or your team to make changes?

8. What else would you like to add to the discussion?
